# A protocol for a systematic review of non-randomised evaluations of strategies to increase participant retention to randomised controlled trials

**DOI:** 10.1186/s13643-018-0696-7

**Published:** 2018-02-20

**Authors:** Adel El Feky, Katie Gillies, Heidi Gardner, Cynthia Fraser, Shaun Treweek

**Affiliations:** Health Services Research Unit, University of Aberdeen, Aberdeen, UK

**Keywords:** Clinical trials, Non-randomised evaluations, Retention strategies, Drop-outs

## Abstract

**Background:**

Randomised control trials are regarded as the gold standard for evaluating the effectiveness and efficacy of healthcare interventions with thousands of trials published every year. Despite significant investment in infrastructure, a staggering number of clinical trials continue to face challenges with retention. Dropouts could lead to negative consequences—from lengthy delays to missing data that can undermine the results and integrity of the trial.

Summarising evidence from non-randomised evaluations of retention strategies could provide complementary information to randomised evaluations that could guide trialists to the most effective ways of increasing retention of participants in clinical trials.

**Methods:**

The following electronic databases will be searched for relevant studies: EMBASE, MEDLINE, the Cochrane Controlled Trials Register, and Cochrane Methodology Register and the search will be limited to English-published studies during the last 10 years to increase relevance to current trials. Non-randomised studies (observational studies) including a comparison of two or more strategies to increase participant retention in randomised trials or comparing one or more strategies with no strategy will be included. The primary outcome will be the proportion of participants remained at the primary analysis as determined in each retention study.

**Discussion:**

This review aims to gather and evaluate evidence on the effect of retention strategies examined in non-randomised studies. It is imperative to collect evidence from obseravational studies to infer whether or not these studies could be considered a practical way to complement or even replace a broadly favourable randomised design. If we find that non-randomised studies to be included in this review are of high quality with adequate control of biases, we will recommend to trialists and others not to rely exclusively on randomised studies and to give meticulous attention to the plentiful evidence that can be obtained from non-randomised studies. Should the results of this review suggest that evaluating retention strategies in observational studies provides insufficient evidence to trialists planning their retention strategies, we will be able to say that there is little point in conducting non-randomised studies and that they would do better to invest their time and resources in a randomised evaluation if possible. Where a non-randomised study design is chosen, the review authors will offer recommendations to trialists and others regarding how to ensure that these studies are conducted in a way that can minimise the risk of bias and increase confidence in the findings.

**Systematic review registration:**

PROSPERO 2017:CRD42017072775.

**Electronic supplementary material:**

The online version of this article (10.1186/s13643-018-0696-7) contains supplementary material, which is available to authorized users.

## Background

Retention of participants is essential to ensure the statistical power and internal validity of clinical trials. High attrition rates reduce power and can bias the estimates of intervention effect, especially if participants lost to follow-up are different from those retained at follow up or if differential attrition is evident between the intervention and control groups in a randomised trial [1]. In a review which evaluated missing outcome data in randomised trials published in four major journals, it was found that 89% of studies reported some missing data and 18% of studies had more than 20% of participants with partly missing outcome data [2]. Recent work with a 2004–2016 cohort of trials funded by the UK Health Technology Assessment Programme reported the median proportion of randomised participants retained to be 89%, meaning that 50% of trials did not have primary outcome data for more than 11% of participants [3].

It is generally accepted that a trial with under 5% loss to follow-up will have a little bias, while missing outcome data from more than 20% may pose a major threat to the validity of the study [4]. Some trial results, however, can be far more vulnerable to missing data than this suggests. The Fragility Index, a way of assessing how fragile a trial conclusion developed by Michael Walsh and colleagues [4] often shows that what is considered statistically significant at *P* < 0.05 can be turned insignificant by a handful of events going in the opposite direction. Crucially, these authors found that for 53% of trials, the number of event swaps needed to change the conclusion was less than the number lost to follow-up. While modest missing data can be handled with statistical methods, the risk of bias can remain [5] and it is impossible to meaningfully fix substantial missing data by statistical means [6].

Attrition is therefore an important and often under-estimated concern for randomised trials, and it is imperative that trialists plan to achieve as close to complete follow-up as possible. Evaluations to explore effective retention strategies have increased recently but still comes a distant second to recruitment in terms of the volume of studies [7]. Different retention strategy themes were identified in three systematic reviews: 1) contact and scheduling methods, 2) characteristics of follow-up visits, 3) non-monetary incentives, 4) monetary incentives, 5) reminders to non-respondents, 6) intensive tracking efforts, 7) description of the study, 8) study benefits, 9) reimbursement and 10) involvement of the community [7–9]. Notwithstanding the knowledge acquired from these reviews, an important limitation is that they did not provide a detailed exploration of retention strategies and potentially disregarded other strategies or themes that may have been evaluated using non-randomised designs. They also did not give much attention to the pre-trial stage, where the likelihood of identifying and addressing future problems is greatest.

The potential contribution that randomised and non-randomised studies can make to the evaluation of effectiveness has provoked considerable controversy [10]. A randomised controlled trial (RCT) is considered as the gold standard design for the assessment of the effectiveness and efficacy of healthcare interventions [11]. Nevertheless, some do not consider RCTs to be the most suitable design to evaluate complex and context-dependent interventions [12, 13]. Several scenarios remain where an RCT may be inappropriate, unnecessary, or impossible [14]. Non-randomised studies, such as controlled and uncontrolled before-after studies, are often undertaken to obtain evidence on the effectiveness of interventions that cannot be randomised, or which are highly unlikely to be tested in randomised studies or where randomisation was simply not considered for one reason or another. In certain circumstances, randomisation may be misleading where the random allocation process may have an influence on the effectiveness of the intervention. Furthermore, experimentation may be practically impossible as investigators may not agree that important uncertainty exists about the relative effectiveness of different interventions and thus regard the study to be unnecessary or unethical.

### The rationale behind conducting this review

Including non-randomised effect evaluations in systematic reviews could be viewed as problematic, particularly when appraising study quality and the likelihood of selection bias and its impact on study results [15]. However, a recent Cochrane review of reviews has shown that there is insufficient evidence of significant effect estimate differences between RCTs and observational studies (79% of the included reviews showed no significant differences between observational studies and RCTs) [16]. Evidence from this Cochrane review suggests that observational studies can be conducted with sufficient rigour to provide complementary evidence or replicate the results of randomised trials. Moreover, we think that the systematic evaluation of what is expected to be a considerable amount of research is crucial (chiefly because without collecting and critically analysing these studies, they are currently disregarded), worthwhile (as there might be significant unknown effects), and will provide tangible results for the trials stakeholders regardless of whether the outcomes support one or more interventions.

To our knowledge, this is the first systematic review that aims to synthesise evidence from non-randomised evaluations of retention strategies in order to supplement existing randomised trial evidence, which together can contribute to optimising rates of retention success in RCTs. It is possible that our review might find that non-randomised retention strategy evaluation has little benefit, in this case, review authors will offer guidance to trialists and others to ensure that these studies are conducted in a way that can be considered comparable to a well-designed randomised study.

### Objectives

- To provide a comprehensive review of retention strategies examined through non-randomised study designs.
- To measure the effect of strategies to promote retention on the number of participants retained in randomised trials and to explore whether the effect varied by trial setting, trial strategy, and retention behaviour (non-return of questionnaires, non-attendance at follow up visits and active withdrawal (i.e. through participant request of no further follow up) from the trial).

## Methods

The systematic review protocol was developed according to the Preferred Reporting Items for Systematic review and Meta-Analysis Protocol (PRISMA-P) guidelines statement, and the PRISMA-P checklist is endorsed (Additional file 1). This protocol will be made publicly available.

### Inclusion criteria

#### Studies to be included

Non-randomised studies comparing two or more strategies to increase participant retention in randomised trials or comparing one or more strategies with no strategy. Non-randomised or observational studies are defined as any quantitative study testing the effectiveness of retention strategies where participants have been allocated to the intervention and control groups by a method that is not random. Eligible non-randomised study designs include observational studies, cohort studies, before-and-after studies, case-control studies, historically controlled studies, uncontrolled longitudinal studies, interrupted time series, and uncontrolled before-and-after studies. The retention trials should be nested in real “host” trials (including feasibility studies) but not hypothetical trials.

#### Participants

Participants from any gender, age, language, cultural, and geographic group will be considered. Studies in healthcare (including different disease areas and all disciplines) and non-healthcare (social sciences, education) topics will be included.

#### Types of interventions

Any strategy designed to optimise retention directed towards the participant, clinician, or researcher will be considered. Trials that include a combination of strategies to improve retention will also be included. The following strategies could be included:

- Strategies that motivate participants or clinicians (e.g. gifts or monetary incentives) with a primary focus on collection of outcome data;
- Strategies that aim to facilitate communication with participants (e.g. telephone follow-up);
- Methodology strategies (e.g. different questionnaire formats or variations in the frequency of follow-up visits);
- Multifaceted strategies (e.g. intensive tracing efforts to locate study participants).
- Strategies that maximise rapport with study participants (e.g. behavioural and motivational strategies).
- Relevance of outcome selection (e.g. disease specific vs generic)

#### Outcome measures

##### Primary outcomes

The primary outcome will be the number of participants retained at the primary analysis point as stated in each retention study. In cases where the time points to measure the primary outcome are not predetermined, the first time point reported will be considered. Where retention at different time points is stated and no definite time point for the primary outcome for the retention study is reported, the closest time point to the intervention in the retention study analyses will be considered.

##### Secondary outcomes

Retention at secondary analysis points and cost of retention strategy per participant.

##### Exclusion criteria

- Studies published before 2007
- Studies where retention strategies are not the primary focus.
- Studies that do not provide retention outcomes.
- Studies that only examine predictors of loss to follow-up.

##### Search strategy

The search strategy was constructed in discussion with an information specialist (CF) with expertise in healthcare databases and systematic reviews. EMBASE, MEDLINE, the Cochrane Controlled Trials Register, and Cochrane Methodology Register will be searched. The search will be limited to English-published studies during the last 10 years to increase relevance to current trials. The search strategy contains both medical subject heading (MeSH) and non-MeSH terms. The search strategy combines text and vocabulary words for concepts such as “retention”, “attrition”, “loss to follow-up”, and “participant dropouts”.

Other search methods will include hand-searching of reference lists of systematic reviews of randomised retention strategies to identify studies that were excluded on account of being not randomised. All retrieved citations will be screened by two independent reviewers to determine eligibility.

The following multifile search strategy for MEDLINE and EMBASE (OVID) will be adapted for the other databases listed.

1. (attrition adj2 (minimi$ or prevent$ or lessen$ or decreas$ or reduc$)).tw.
2. (drop$-out$ adj2 (minimi$ or prevent$ or lessen$ or decreas$ or reduc$)).tw.
3. (dropout$ adj2 (minimi$ or prevent$ or lessen$ or decreas$ or reduc$)).tw.
4. (strateg$ adj2 (dropout$ or drop$-out$)).tw.
5. ((lost or loss) adj2 (follow-up or followup)).tw.
6. (withdrawl$ adj2 (minimi$ or prevent$ or lessen$ or decreas$ or reduc$)).tw.
7. (strateg$ adj2 (attrition or followup or follow-up)).tw.
8. (retention adj5 (increas$ or encourag$ or maximi$ or promot$ or improv$ or influenc$ or success$)).tw.
9. (compliance adj2 (increas$ or encourag$ or maximi$ or promot$ or improv$)).tw.
10. (strateg$ adj2 response$).tw.
11. (questionnaire$ adj3 respon?e$ adj2 (strateg$ or increas$ or encourag$ or maximi$ or promot$ or improv$)).tw.
12. (retention adj1 rate$).tw.
13. (attrition adj1 rate$).tw.
14. (follow up adj1 rate$).tw.
15. (retention adj3 (strateg$ or intervention? or method$ or technique$)).tw.
16. (compliance adj3 (strateg$ or intervention? or method$ or technique$)).tw.
17. (questionnaire$ adj3 response$ adj2 (method$ or technique$)).tw.
18. ((incentive$ or reminder$ or method$) adj3 (Retention or respon?e$)).tw.
19. (difficult$ adj2 (retain$ or retention)).tw.
20. (retention adj3 (participant? or subject? or patient?)).tw.
21. ((increase or maintain$) adj3 (partipa$ or respon?e$ or compliance)).tw.
22. Patient Dropouts/use ppez
23. Patient Dropout/use emef
24. or/1–23
25. research subjects/use ppez
26. research subject/use emef
27. exp. Clinical Trials as Topic/use ppez
28. exp. “clinical trial (topic)”/use emef
29. Observational Study as Topic/
30. ((research or trial? or study or studies or pilot or program$ or longitudinal or prospective or retrospective) and (attrition or drop$ out$ or dropouts or withdrawl$ or follow up or retention or retain$ or compliance or participation or recruit$ or engag$)).ti.
31. or/25–30
32. 24 and 31
33. (letter or editorial or comment or note or abstract).pt.
34. 32 not 33
35. limit 34 to english language
36. limit 35 to yr. = “2010–2017” [Embase 3890 MEDLINE 3085]
37. limit 36 to yr. = “2010–2014”
38. remove duplicates from 37
39. limit 36 to yr. = “2015–2017”
40. remove duplicates from 39
41. 38 or 40

##### Data management

The EndNote reference management software will be used to manage the search results, and the EndNote de-duplication tool will be applied to remove any duplicate records. An Excel spreadsheet will be used to track all inclusions/exclusions that will help us to develop a Preferred Reporting Items for Systematic Reviews and Meta-Analyses (PRISMA) flow diagram when the screening process is carried out.

##### Identification of eligible studies

The abstracts of all records retrieved from the search will be screened by two independent reviewers. The full-text check will be carried out for all potentially eligible studies by two independent review authors. Where necessary, study authors will be contacted to seek information that will resolve any questions regarding the eligibility of studies. Any disagreements among review authors will be discussed and resolved. Where necessary, a third reviewer will be involved to adjudicate unresolved disagreements.

##### Data extraction

Two reviewers will independently extract information from each of the included studies using a standardised data extraction form designed for the purpose of the review. Data to be extracted for the host trial will be objective, trial setting, clinical area, and comparators. For the nested retention study, data for start time relative to the host trial, number of participants, objective, primary outcome, and method of follow-up will be extracted. Details of the retention intervention will include type, timing and frequency of administration, method of allocation, numbers allocated to groups and retained at primary analysis, and data necessary to assess the risk of bias. Retention strategies and retention rates at different follow-up time points will be extracted independently. Any disagreement will be discussed and resolved. Where necessary, a third reviewer will be involved to adjudicate unresolved disagreements. Where required, corresponding authors will be contacted to seek additional information.

##### Quality assessment of included studies

The Cochrane ROBINS-I (“Risk Of Bias In Non-randomised Studies - of Interventions”) [17] tool will be used to appraise the quality of included studies. The tool offers a structured and comprehensive approach for the assessment of non-randomised studies of interventions. Central features of ROBINS-I include the use of signalling questions to guide risk of bias judgements within seven bias domains. The quality assessment will be carried out by two review authors. Any disagreement will be discussed and resolved. Where necessary, a third reviewer will be involved to adjudicate unresolved disagreements. Where necessary, study authors will be contacted for additional information to clarify study methods.

##### Assessment of heterogeneity

Where study population, intervention, and outcome data are adequately similar to justify pooling the results in a meta-analysis, visual evidence of heterogeneity in forest plots will be investigated together with statistical evidence of heterogeneity using the chi-square statistic, and the *I*^2^ test will be used to quantify the degree of heterogeneity [18]. Where significant heterogeneity is found (*I*^2^ ≥ 50%), informal explanations will be conducted and the random-effect model will be used to summarise data where appropriate.

##### Data analysis and synthesis

If the included studies are statistically homogeneous (*I*^2^ < 50%), a meta-analysis using a fixed-effect model will be performed. Otherwise, the random-effect model will be employed. The causes of heterogeneity will be evaluated to supplement choice of the model using the Cochrane Handbook for Systematic Reviews of Interventions Version 5.1.0 [19].

It is anticipated that there will be much diversity in the included studies. Where it is not appropriate to perform a meta-analysis, included studies will be combined in a narrative synthesis. To ensure the synthesis is a rigorous and transparent process, review authors will meet to discuss and categorise different retention strategies from the included studies. Firstly, reviewers will review the strategies independently and assign each retention strategy to the relevant category. The independent results will then be discussed and differences will be reconciled before a final list of major retention categories is provided.

##### Measures of the effect

For dichotomous outcomes (retention or attrition rates), risk ratios and their 95% confidence intervals will be calculated to determine the effect of strategies on participant retention. It is not clear how participant benefits or difficulties with strategies will be measured, so the data available will be examined and then the most appropriate effect measure will be determined.

##### Unit of analysis issues

It is anticipated that most of the included studies will be before-and-after studies with the individual participant as the unit of analysis. We will only include clustered studies in the meta-analysis if sufficient data were reported to allow inclusion of analyses that adjusted for clustering; an odds ratio (OR) will be used in the summary effect of the meta-analysis result if the risk difference or risk ratio clustering adjusted analyses were not possible with available data.

##### Handling missing data

The amount and reasons for missing data will be recorded. Every effort will be made to contact study authors for data essential to appraise the quality of included studies, numbers allocated to each group, and number of participants retained at the primary endpoint. When assessing the risk of bias, drop-outs will be reported and considered as a potential source.

##### Assessment of reporting bias

Where sufficient data will be available (10 or more studies of the same retention strategy, study population, and outcomes)**,** tests for funnel plot asymmetry will be used to investigate reporting bias.

##### Assessment of the quality of evidence

The Grading of Recommendations Assessment Development and Evaluation (GRADE) system will be used to rate the certainty of evidence from the included studies [20]. Evidence from robust non-randomised studies will be generally graded as low quality. Nevertheless, if the effect yielded by such studies is large enough and there is no clear evidence of bias to explain those effects, the evidence might be rated as moderate or even high quality. GRADE assessments will be applied independently by two reviewers to judge the certainty of the evidence.

## Discussion

This review will gather and evaluate evidence on the effect of retention strategies examined in non-randomised studies. It is clear that many researchers are somewhat dichotomised concerning whether to rely solely on randmoised study designs while searching for evidence on effective retention strategies or to also consider non-randomised studies as a reliable surrogate to randomised studies when conducted with sufficient rigour. Recent evidence suggests that high-quality non-randomised studies can provide outcomes that are similar to those found in randomised studies and that study quality may have a greater impact on treatment effect size than randmisation alone, denoting that randmisation should not be considered as a sound proxy for overall trial quality [16]. Therefore, it is imperative to collect evidence from observational studies to infer whether or not these contentious studies could be considered a practical way to complement or even replace a randomised design in some cases. If we find that non-randomised studies to be included in this review are of high quality with adequate control of biases, we will recommend to trialists and others not to rely exclusively on randomised studies and to direct their attention to the plentiful evidence that can be generated from non-randomised evaluations. If the included studies are of low quality with inappropriate control of confounding factors that might threaten the validity of findings, review authors will be able to recommend to funders and trial stakeholders that researchers should only consider conducting observational studies of high methodological quality that provide results that can be part of an evidence base to inform trial design decisions. Furthermore, the review might introduce innovative and promising retention strategies that need to be tested in a more rigorous randomised study.

## Additional file

Additional file 1: There are no additional titles or legends to be included in this systematic review protocol. The PRISMA checklist is endorsed and has been uploaded as a table file. (DOCX 18 kb)

## Abbreviations

EMBASE: Excerpta Medica dataBASE
GRADE: Grading of Recommendations Assessment, Development and Evaluation
MEDLINE: Medical Literature Analysis and Retrieval System Online
MeSH: Medical subject heading
OR: Odds ratio
PRISMA-P: Preferred Reporting Items for Systematic Review and Meta-Analysis Protocol
RCT: Randomised control trial
ROBINS-I: Risk of Bias in Non-Randomised Studies - of Interventions
